# Supplementary material for: Role of Survivin in Bladder Cancer: Issues to Be Overcome When Designing an Efficient Dual Nano-Therapy
Source: Pharmaceutics. 2021 Nov 19;13(11):1959. doi: 10.3390/pharmaceutics13111959 (PMC8618611; doi:10.3390/pharmaceutics13111959)
Supplement: Supplementary file 1 [file pharmaceutics-13-01959-s001.zip › pharmaceutics-1406835-SI.pdf]

pharmaceutics

Supplementary Materials: Role of Surviving in Bladder Cancer: Issues to Be Overcome When Designing an Efficient Dual Nano-Therapy

Maria Arista-Romero, Anna Cascante, Cristina Fornaguera and Salvador Borrós

Table S1. DoE planning. Set up of the experiments performed combining the selected factors (7) and giving 2 levels to each them.

	siRNA (mg/mL)	Polymer	Buffer (M)	Temperature	Incubation (min)	n/p Ratio
1	0.03	RK	25 mM	25ºC	30 min	100:1
2	0.01	RK	25 mM	37ºC	10 min	150:1
3	0.01	R3	25 mM	37ºC	30 min	100:1
4	0.03	R	10 mM	37ºC	30 min	150:1
5	0.01 l	RK	10 mM	25ºC	30 min	150:1
6	0.03	R	25 mM	25ºC	10 min	150:1
7	0.03 l	RK	10 mM	37ºC	10 min	100:1
8	0.01 l	R	10 mM	25ºC	10 min	100:1

In this Table, the factors selected for the design of experiments were combined in the detailed way to perform 8 experiments and determine the effect of each factor on the results.

Table S2. DoE results. Initial (0 min) and final size (60 min) and PDI of the resulting nanoparticles in all 8 experiences from the experience study.

	Size		PDI	
Experience	Initial	Final (60 min)	Initial	Final (60 min)
1	98.59	101.26	0.21	0.29
2	80.81	1632.6	0.31	0.66
3	388.13	1198.3	0.32	0.63
4	92.41	231.4	0.29	0.47
5	119.1	187.5	0.27	0.36
6	503.76	1736.6	0.35	0.92
7	113.37	441.8	0.42	0.54
8	434.46	1253.6	0.3	0.66

In the Table, size and PDI were evaluated following the conditions of the DoE, just after NP preparation and after 60 min incubation. Selected experiences are labelled in green (selected based on their size, PDI and stability).

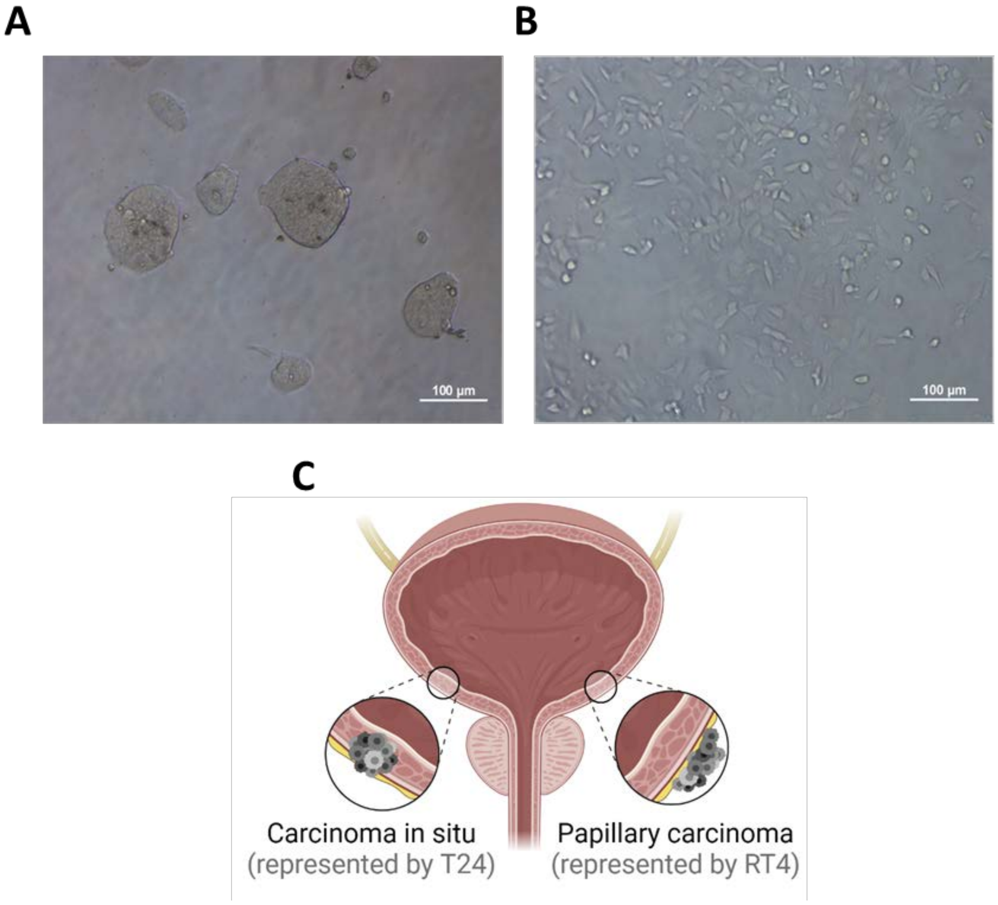

Figure S1. Cell micrographics. A – RT4 and B – T24 cell lines; and C – schematic representation of the bladder tumor types.

Two cell lines, RT4 and T24, were used to perform the experiments, since they represent a papillary papilloma and a carcinoma, respectively, which are structured differently in patients, as represented in Figure S1.C.

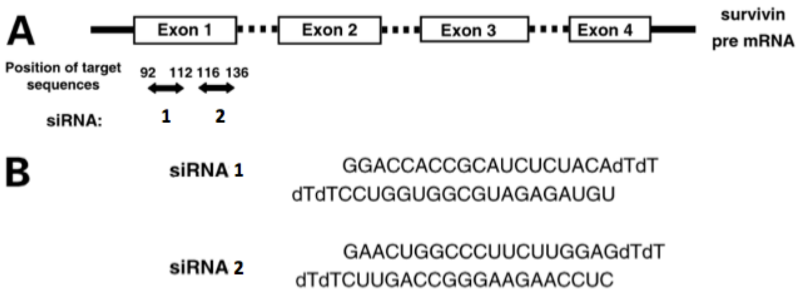

Figure S2. A- Schematic representation of the survivin pre-mRNA and location of each target sequence in survivin mRNA (Genbank accession no. NM_001168.1). B – Nucleotide sequences of siRNAs. (Paduano F. 2006).

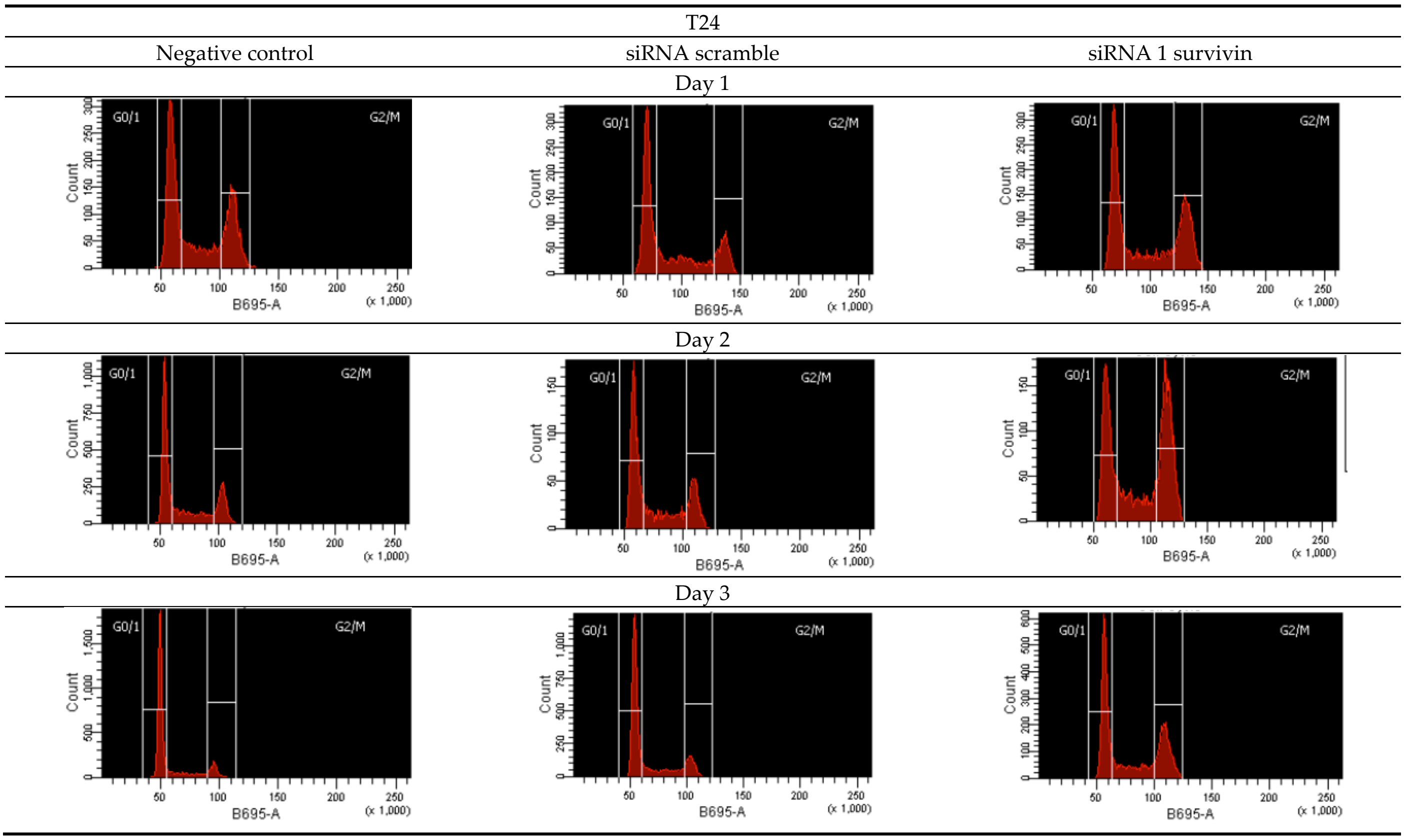

Figure S3. T24 cell cycle interference of siRNAs anti-survivin. Study over the time on the cell cycle phases as a function of the treatment administered to cells.

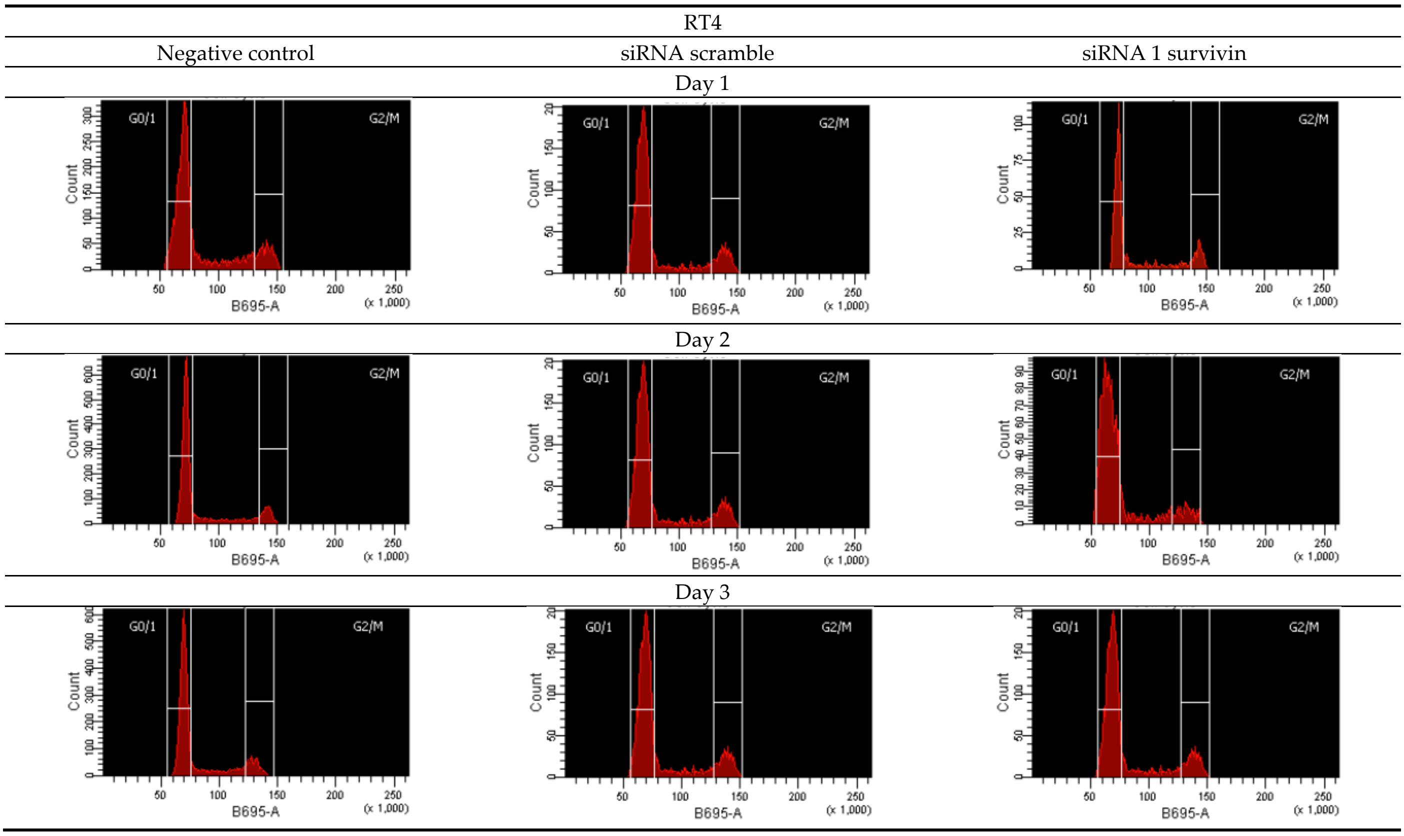

Figure S4. RT4 cell cycle interference of siRNAs anti-survivin. Study over the time on the cell cycle phases as a function of the treatment administered to cells.

As shown in Figures S3 and S4, the cell cycle was studied over time with samples treated with siRNA scramble and siRNA1 against survivin. Basically, cells were in G0/1, showing a normal progression over time.

When T24 cells were treated with siRNA1 against survivin, it could be seen that after one day of transfection, cells started to stay in more affluence in G2/M. This effect was more obvious in the day 2 of transfection when cells were mainly concentrated on the stage of G2/M. The day 3 after transfection this effect was decreased but still evident.

A

B

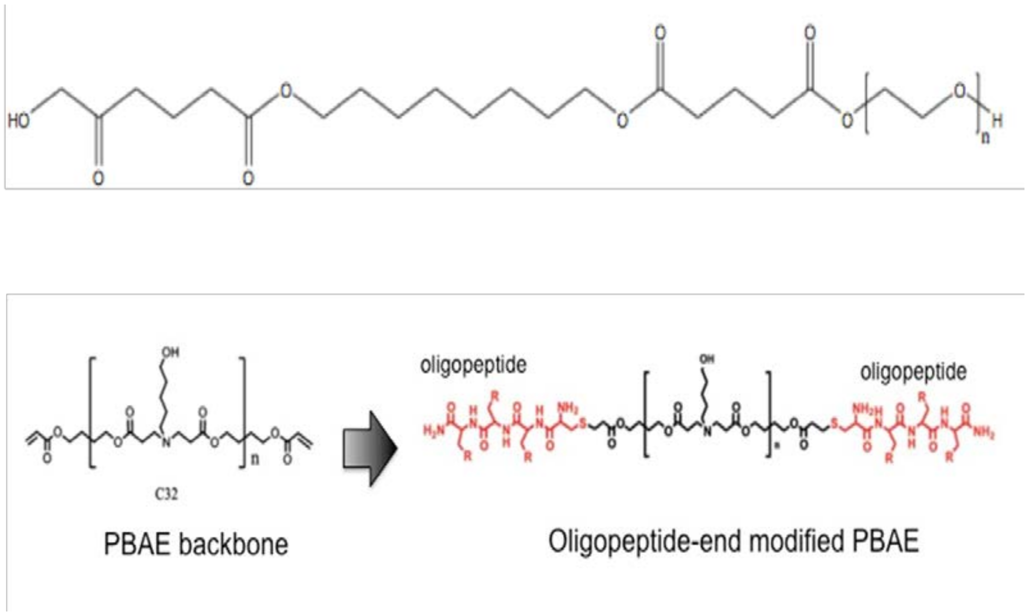

Figure.S5. Polymers used to engineer both monotherapies. A – P polymer structure; and B – pBAE structure. P polymer was used for the encapsulation of PTX, while pBAE was used for survivin siRNA encapsulation. Both are polymers proprietary for our research groups. In both cases, although not mentioned here, we have a library of polymers with slight modifications to give added functionalities to resulting nanoparticles, such as hydrophobicity, active targeting and stability.
